# Pre-transplant MRD negativity predicts favorable outcomes of CAR-T therapy followed by haploidentical HSCT for relapsed/refractory acute lymphoblastic leukemia: a multi-center retrospective study

**DOI:** 10.1186/s13045-020-00873-7

**Published:** 2020-05-04

**Authors:** Houli Zhao, Jieping Wei, Guoqing Wei, Yi Luo, Jimin Shi, Qu Cui, Mingfeng Zhao, Aibin Liang, Qing Zhang, Jianmin Yang, Xin Li, Jing Chen, Xianmin Song, Hongmei Jing, Yuhua Li, Siguo Hao, Wenjun Wu, Yamin Tan, Jian Yu, Yanmin Zhao, Xiaoyu Lai, Elaine Tan Su Yin, Yunxiong Wei, Ping Li, Jing Huang, Tao Wang, Didier Blaise, Lei Xiao, Alex H. Chang, Arnon Nagler, Mohamad Mohty, He Huang, Yongxian Hu

**Affiliations:** 1 Bone Marrow Transplantation Center, The First Affiliated Hospital, School of Medicine, Zhejiang University, No.79 Qingchun Road, Hangzhou, China; 2 Zhejiang Province Engineering Laboratory for Stem Cell and Immunity Therapy, Hangzhou, China; 3 Institute of Hematology, Zhejiang University, Hangzhou, China; 4 Department of Hematology, Beijing Tiantan Hospital, Capital Medical University, Beijing, China; 5 Department of Hematology, Tianjin First Central Hospital, Tianjin, China; 6 Department of Hematology, Shanghai Tongji Hospital, Shanghai, China; 7 Department of Hematology, Guangdong Second Provincial General Hospital, Guangzhou, China; 8 Department of Hematology, Changhai Hospital of Shanghai, Shanghai, China; 9 Department of Hematology, Xiangya Third Hospital, Changsha, China; 10 Department of Hematology, Shanghai Children’s Medical Center, Shanghai, China; 11 Department of Hematology, Shanghai General Hospital, Shanghai, China; 12 Department of Hematology, Peking University Third Hospital, Beijing, China; 13 Department of Hematology, Zhujiang Hospital of Southern Medical University, Guangzhou, China; 14 Department of Hematology, Xinhua Hospital of Shanghai, Shanghai, China; 15 Institut Paoli-Calmettes, Marseille, France; 16 Innovative Cellular Therapeutics Co, Ltd, Shanghai, China; 17 Shanghai YaKe Biotechnology Ltd, Shanghai, China; 18 Hematology and Bone Marrow Transplantation Division, Chaim Sheba Medical Center, Tel-Hashomer, Israel; 19 Sorbonne University, Saint-Antoine Hospital, INSERM UMRs 938, Paris, France

**Keywords:** Chimeric antigen receptor T cell therapy, Haploidentical hematopoietic stem cell transplantation, Leukemia-free survival, Minimal residual disease negativity, Overall survival, Relapsed/refractory acute lymphoblastic leukemia

## Abstract

**Background:**

Consolidative allogeneic hematopoietic stem cell transplantation is a controversial option for patients with relapsed/refractory acute lymphoblastic leukemia after chimeric antigen receptor T cell (CAR-T) therapy. We performed a multicenter retrospective study to assess whether patients can benefit from haploidentical hematopoietic stem cell transplantation after CAR-T therapy.

**Methods:**

A total of 122 patients after CAR-T therapy were enrolled, including 67 patients without subsequent transplantation (non-transplant group) and 55 patients with subsequent haploidentical hematopoietic stem cell transplantation (transplant group). Long-term outcome was assessed, as was its association with baseline patient characteristics.

**Results:**

Compared with the non-transplant group, transplantation recipients had a higher 2-year overall survival (OS; 77.0% versus 36.4%; *P* < 0.001) and leukemia-free survival (LFS; 65.6% versus 32.8%; *P* < 0.001). Multivariate analysis showed that minimal residual disease (MRD) positivity at transplantation is an independent factor associated with poor LFS (*P* = 0.005), OS (*P* = 0.035), and high cumulative incidence rate of relapse (*P* = 0.045). Pre-transplant MRD-negative recipients (MRD− group) had a lower cumulative incidence of relapse (17.3%) than those in the non-transplant group (67.2%; *P* < 0.001) and pre-transplant MRD-positive recipients (MRD+ group) (65.8%; *P* = 0.006). The cumulative incidence of relapse in MRD+ and non-transplant groups did not differ significantly (*P* = 0.139). The 2-year LFS in the non-transplant, MRD+, and MRD− groups was 32.8%, 27.6%, and 76.1%, respectively. The MRD− group had a higher LFS than the non-transplantation group (*P* < 0.001) and MRD+ group (*P* = 0.007), whereas the LFS in the MRD+ and non-transplant groups did not differ significantly (*P* = 0.305). The 2-year OS of the MRD− group was higher than that of the non-transplant group (83.3% versus 36.4%; *P* < 0.001) but did not differ from that of the MRD+ group (83.3% versus 62.7%; *P* = 0.069). The OS in the non-transplant and MRD+ groups did not differ significantly (*P* = 0.231).

**Conclusion:**

Haploidentical hematopoietic stem cell transplantation with pre-transplant MRD negativity after CAR-T therapy could greatly improve LFS and OS in patients with relapsed/refractory acute lymphoblastic leukemia.

**Trial registration:**

The study was registered in the Chinese clinical trial registry (ChiCTR1900023957).

## Background

Patients with relapsed/refractory acute lymphoblastic leukemia (R/R ALL) usually have a very poor prognosis with an expected median survival of less than 6 months, and the overall survival (OS) at 5 years is only 5-10% [1]. Complete remission (CR) rates after the first salvage chemotherapy are approximately 30-46%, and these rates drop sharply to 18-25% after the second salvage chemotherapy. Allogeneic hematopoietic stem cell transplantation (allo-HSCT) is a potentially curative option for hematological malignancies and has improved the prognosis of R/R ALL over the past two decades. However, for patients who failed to achieve minimal residual disease (MRD) negativity before allo-HSCT, their 3-year OS and leukemia-free survival (LFS) were only 23.5% and 20.6%, respectively [2]. Thus, novel therapeutic strategies to improve prognosis of these patients are urgently needed.

Recently, chimeric antigen receptor T cells (CAR-Ts) targeting CD19 or CD22 have been reported to successfully improve treatment outcomes for R/R ALL [3, 4]. In our previous clinical trial of CD19-targeted CAR-T therapy against R/R ALL, a CR rate of 92.3% was achieved [5]. However, data from long-term follow-ups in CAR-T trials show that relapse after CAR-T treatment still remains a predominant obstacle. Relapse rates of 20–70% were described when the follow-up period was sufficiently long [6]. The median LFS in patients with a low disease burden was 10.6 months, whereas that in patients with a high disease burden was 5.3 months [3]. With respect to the high relapse rates and the potentially unique relapse mechanisms after CAR-T treatment, consolidation therapy following CAR-T treatment may be considered as a necessary strategy, to reduce the risk of relapse and to maintain the status quo of CR.

Notably, consolidative allo-HSCT after CAR-T therapy is still a controversial option for improving long-term LFS. Park et al. reported that of seventeen patients who underwent allo-HSCT after CAR-T therapy [3]. Relapse and transplantation-associated complications were the main causes of death for those who received CAR-T therapy before allo-HSCT, and the patients seemed not to benefit from allo-HSCT after CAR-T treatment [3]. Hay et al. found that allo-HSCT after CD19 CAR-T cell therapy was associated with a better LFS; however, they also reported a better LFS in MRD-negative CR patients who proceeded to allo-HSCT than those who did not [7]. In addition to allo-HSCT from conventional donors, considerable progress has been made regarding haploidentical HSCT (haplo-HSCT) in recent years, and clinical outcomes of patients receiving haplo-HSCT have been reported to be similar to those receiving HLA-matched HSCT [8]. To date, the efficacy and safety profiles of haplo-HSCT after CAR-T treatment have not been assessed. Regarding the efficacy and transplant-associated complications, whether patients would benefit from allo-HSCT after CAR-T treatment remains controversial. And, no pre-transplant biomarker has been recommended to predict outcomes after transplantation. Further researches are needed to determine effectiveness of allo-HSCT consolidation therapy and to assess factors affecting long-term clinical outcomes.

Therefore, we designed a multicenter retrospective study to assess the efficacy and safety profiles of CAR-T therapy alone or CAR-T therapy followed by haplo-HSCT in patients with R/R ALL. We also determined potential prognostic factors associated with clinical outcomes in these patients.

## Methods

### Patients

**Fig. 1:**
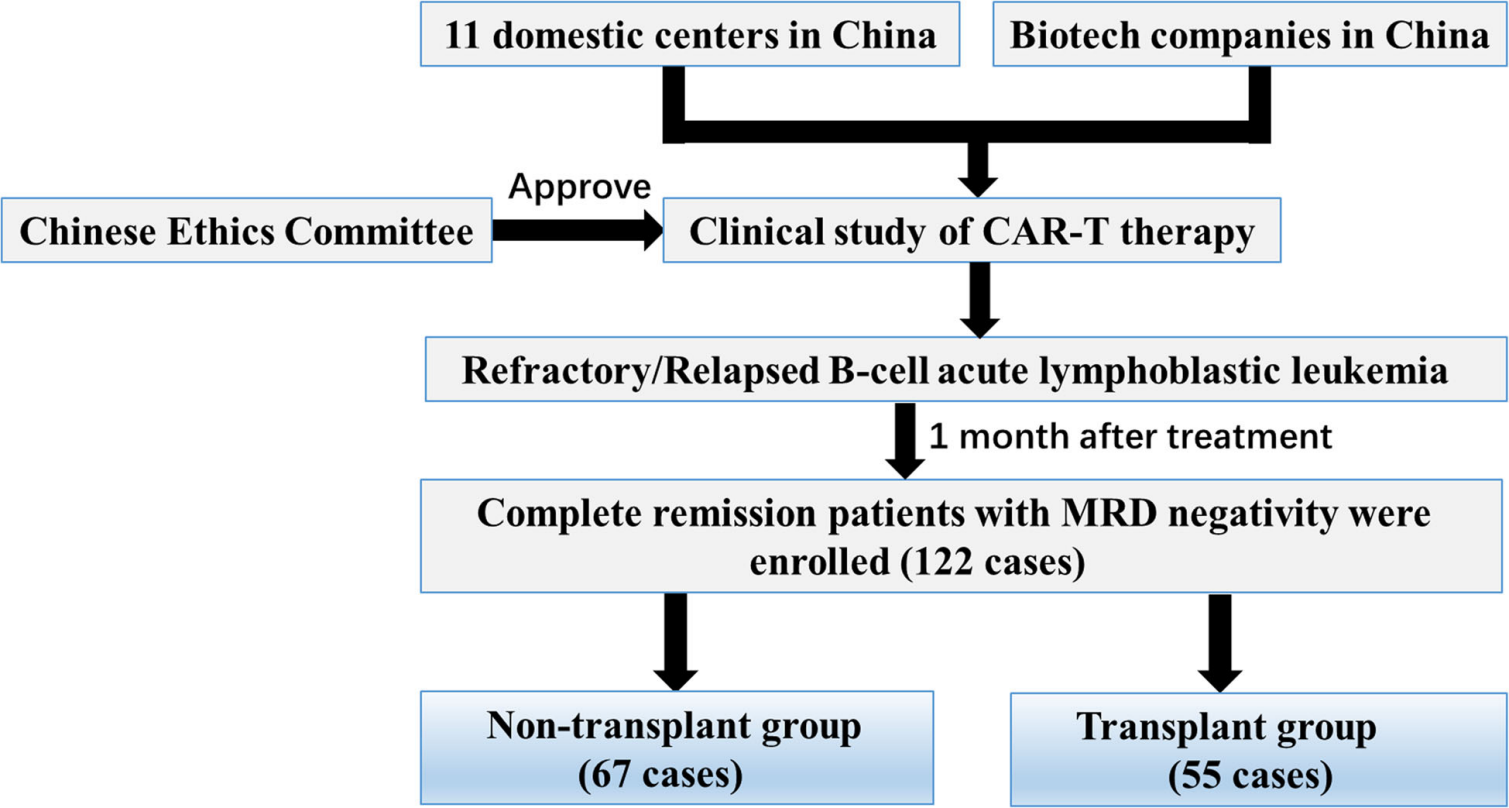
Patient enrollment flow chart

This multicenter retrospective study included patients undergoing CAR-T treatment selectively followed by haplo-HSCT at 11 domestic centers in China from July 2015 to December 1, 2019 (Supplementary Table 1; Fig. 1). Patient inclusion criteria were as follows: (1) age less than 70 years old; (2) relapsed or refractory CD19-positive ALL before CAR-T treatment; and (3) CD19 CAR-T treatment followed by achievement of MRD negativity in CR. Exclusion criteria included (1) pregnancy and lactation; (2) having conditions such as central nervous system diseases, clinically significant cardiovascular diseases, severe hepatic and renal dysfunctions, and various active infections; (3) having received systemic steroids in the previous 2 weeks (except for inhaled steroids) or gene therapies; (4) having any other conditions that might increase treatment risks; and (5) receiving CAR-T therapy followed by HLA-matched related HSCT or unrelated HSCT. The study was approved by the ethics review committee of each institution and retrospectively registered in the Chinese clinical trial registry (www.chictr.org.cn/showproj.aspx?proj=39004) (ChiCTR1900023957) on June 19, 2019. All participants provided written informed consent in accordance with the Declaration of Helsinki.

### Treatment protocol

Anti-CD19 chimeric antigen receptor T cells constructed with 4-1BB costimulatory domain were generated via lentiviral vector from fresh leukapheresis material by Chinese biotech companies following the same manufacture standard as previously reported [5, 9]. All the CAR-T cells required quality control according to the Code of manufacturing quality management for Chimeric Antigen Receptor T Cells (CAR-T cells) based-medicinal product formulated by China Medicinal Biotech Association [10] before discharge. After a lymphocyte-depleting chemotherapy with a fludarabine-cyclophosphamide regimen, patients received an infusion of CD19 CAR-T cells. Response to therapy was assessed using morphological analysis and 6-color flow cytometry including CD10, CD19, CD20, CD34, CD38, and CD45. A sensitivity of 0.01% for MRD was achieved in all samples analyzed. Patients were then followed-up in out-patient departments for MRD detection (using flow cytometry) 1, 2, 3, 6, 12, 18, 24, 36, and 48 months after CAR-T therapy or haplo-HSCT. Depending on the willingness, economic background, and quality of life of patients, some patients received haplo-HSCT after CAR-T infusion. Patients who were going to receive the HSCT should provide MRD report within 1 week assessed by 6-color flow cytometry. Patients were divided into two groups: CAR-T treatment without transplant (non-transplant group) and CAR-T treatment followed by haplo-HSCT (transplant group).

Conditioning regimens included myeloablative regimens (busulfan and cyclophosphamide based and total body irradiation based) or nonmyeloablative regimens, as previously reported [11–14]. Most patients were younger, fit, and eligible for myeloablative conditioning. Patients eligible for myeloablative conditioning were typically older (depending on the center and time period, aged more than 60 years). After the conditioning regimen, three patients received peripheral blood stem cells combined with bone marrow stem cells, and 54 patients received peripheral blood stem cells from haplo-identical donors. Graft-versus-host-disease (GVHD) prophylactic regimens were determined by the individual transplant physician based upon disease-related and transplant-related considerations.

### Study end points, definitions, and statistical analysis

The study aimed to assess the safety and efficacy of CAR-T therapy alone or CAR-T therapy followed by haplo-HSCT for the treatment of R/R ALL. The primary end point was OS and LFS in patients with different pre-transplant MRD status. The secondary endpoints included cumulative incidences of cytokine release syndrome (CRS), relapse, non-relapse mortality (NRM), acute GVHD (aGVHD), chronic GVHD (cGVHD), and viral infection after haplo-HSCT. For estimation of LFS, MRD-positive patients were not considered as having relapsed. MRD positivity was defined as having disease at a threshold of more than one ALL cell per 10,000 nuclear cells in the bone marrow, and LFS was defined as time to relapse or death, whichever occurs first. All measurement data were described using median and range and compared using *t* tests. Enumeration data were presented as frequency (%) and compared using chi-square tests. A competitive risk model was used to estimate the 100-day cumulative incidences of cGVHD requiring systemic steroid therapy and grade III-IV aGVHD and 2-year cumulative incidences of Epstein–Barr virus (EBV) or cytomegalovirus (CMV) infection and relapse. Follow-up time was estimated using the reverse Kaplan-Meier method, whereas OS and LFS were estimated using the Kaplan-Meier method. A Cox regression model was used to obtain the hazard ratio (HR) estimates and corresponding 95% confidence intervals (CI) for OS and LFS. All *P* values were two-sided, and results were considered statistically significant at *P* < 0.05. Data were analyzed using IBM SPSS Statistics 24 and R version 3.4.3.

## Results

### Patient characteristics

A total of 122 patients were enrolled in the study. The number of patients included in the non-transplant and transplant groups was 67 and 55, respectively (Fig. 1). Baseline characteristics are summarized in Table 1. The median age at the time of CAR-T therapy was 27 and 26 years in the non-transplant group and transplant group, respectively. Thirty-eight (31.1%) patients were primary refractory to chemotherapy, and 26 (20.3%) patients had previously undergone allo-HSCT. Twenty-five (20.5%) patients were Philadelphia chromosome/*BCR-ABL* positive (Ph+) prior to CAR-T therapy. Prior to lymphodepletion, 87 (71.3%) patients had morphological disease (≥ 5% blasts) in the bone marrow, with a median blast cell percentage of 33.8%. There were only significant differences between the two groups in terms of prior allo-HSCT and source of CAR-T cells. The fraction of patients who had previously undergone allo-HSCT was higher in the non-transplant group (22/67) than in the transplant group (3/55) (*P* < 0.001). Accordingly, the fraction of patients who received autologous CAR-T cells was higher in the transplant group (50/55) than in the non-transplant group (44/67) (*P* = 0.002). Multivariate analysis models for clinical outcome included these two variables and the variable “bridging to haplo-HSCT after CAR-T therapy.” Neither prior allo-HSCT nor source of CAR-T cells were associated with clinical outcome, but bridging to haplo-HSCT after CAR-T therapy was associated with long LFS (HR: 0.244, 95% CI: 0.136-0.437; *P* < 0.001) and OS (HR: 0.275, 95% CI: 0.142-0.531; *P* < 0.001) as well as low cumulative incidence of relapse (HR: 0.163, 95% CI: 0.081-0.329; *P* < 0.001). In the transplant group, 15 patients (27.3%) showed pre-transplant MRD positivity. There were no statistically significant differences with respect to median age, gender, chromosomal aberrations, and gene mutations between the two groups (*P* > 0.05; Table 1).

**Table 1:** Patient characteristics between non-transplant group and transplant group

			*P*
Characteristics	**Non-transplant (*n*= 67)**	**Transplant (*n* = 55)**	**Non-transplant vs transplant**
Gender, *n* (%)			0.153
Male	34 (50.7)	35 (63.6)	
Female	33 (49.3)	20 (36.4)	
Age, years			0.332
Median (range)	27 (9, 65)	26 (3, 65)	
Hyperploidy, *n* (%)	3 (4.5)	3 (5.5)	0.804
Hypoplodiy, *n* (%)	1 (1.5)	1 (1.8)	0.888
Complex karyotype, *n* (%)	4 (6.0)	2 (3.6)	0.553
iAMP 21^a^, *n* (%)	1 (1.5)	1 (1.8)	0.888
ETV6-RUNX1, *n* (%)	1 (1.5)	0 (0)	0.363
E2A-PBX1, *n* (%)	1 (1.5)	0 (0)	0.363
MYC mutation, *n* (%)	1 (1.5)	0 (0)	0.363
HOX11 mutation, *n* (%)	1 (1.5)	0 (0)	0.363
KMT2A rearranged, *n* (%)	3 (4.5)	5 (9.1)	0.306
BCR-ABL1, *n* (%)	11 (16.4)	11 (20.0)	0.609
Ph like, *n* (%)	1 (1.5)	4 (7.3)	0.109
IgH rearranged, *n* (%)	1 (1.5)	0 (0)	0.363
IKZF1 mutation, *n*%	3 (4.5)	7 (12.7)	0.098
Poor-risk cytogenetics^b^, *n* (%)	18 (26.9)	21 (38.2)	0.182
Good-risk cytogenetics^c^, *n* (%)	2 (3.0)	2 (3.6)	0.841
Primary refractory to chemotherapy, *n* (%)	17 (25.4)	21 (38.2)	0.128
Total number of relapses before CAR-T			0.405
Median (range)	1 (0, 6)	1 (0, 3)	
Prior lines of therapy			0.888
Median (range)	4 (2, 15)	3 (2, 9)	
Prior allo-HSCT, *n* (%)	22 (32.8)	3 (5.5)	**< 0.001**
Extramedullary infiltration, *n* (%)	9 (13.4)	3 (5.5)	0.141
Platelet count before CAR-T, 10^9^/L			0.339
Median (range)	130 (15, 412)	140 (12, 389)	
PLT < 1*LLN before CAR-T, *n* (%) (*n* = 66 + 52)	28 (42.4)	15 (28.8)	0.128
LDH > 1*ULN before CAR-T, *n* (%) (*n* = 66 + 52)	26 (39.4)	25 (48.1)	0.345
Blast cells in bone marrow before CAR-T			0.766
Median (range)	22.5% (0, 97.0%)	16.0% (0, 90.0%)	
CAR-T cell dose, 10^6^/kg			0.853
Median (range)	3.0 (0.3, 25.0)	5.0 (0.1, 30.4)	
Source of CAR-T cells, *n* (%)			**0.002**
Autologous	44 (65.7)	50 (90.9)	
Recipient-derived allogenic	15 (22.4)	3 (5.5)	
Donor-derived allogenic	7 (10.4)	0 (0)	
Third-party	1 (1.5)	2 (3.6)	

^a^*iAMP21* intrachromosomal amplification of chromosome 21

^b^Poor-risk cytogenetics are defined as hypodiploidy, KMT2A rearranged, t(v;14q23)/IgH rearranged, t(9;22)(q34;q11.2): BCR-ABL1, complex karyotype, Ph-like, and iAMP21

^c^Good-risk cytogenetics are defined as hyperdiploidy, and t(12;21)(p13;q22): ETV6-RUNX1

### CAR-T therapy-associated toxicities

The most common CAR-T therapy-associated toxicity was CRS. The peak of CRS occurred in a median time of 6 days after CAR-T infusion. Complicated CRS of grades 0, 1–3, and 4–5 was observed in 9, 108, and 5 patients, respectively. Platelet count below 10^4^/μL with a median onset time of 2 days after CAR-T therapy occurred in 77 (63.1%) patients, and the condition lasted for a median time of 8 days. Neutropenia occurred at a median time of 2 days after CAR-T infusion in 91 (74.6%) patients and lasted for a median time of 5 days after onset (Supplementary Table 2). Except for the 9 patients with grade 0 CRS, patients (*n* = 113) suffered from pyrexia and were monitored by examination of bacterial and fungal cultures. Three patients had septicemia due to *Burkholderia cepacia*, *Trichosporon asahii*, and *Klebsiella pneumoniae*, whereas three patients suffered from pneumonia, with *Stenotrophomonas maltophilia*, alpha streptococcus/*Neisseria pharyngis*, and *Klebsiella pneumoniae* infections in the sputum. *Salmonella bovismorbificans* and *Candida albicans* infections in the stools were recorded in one patient with diarrhea. There were no statistically significant differences with respect to factors associated with CAR-T therapy between the two groups (Supplementary Table 2).

### Long-term clinical outcomes

**Fig. 2:**
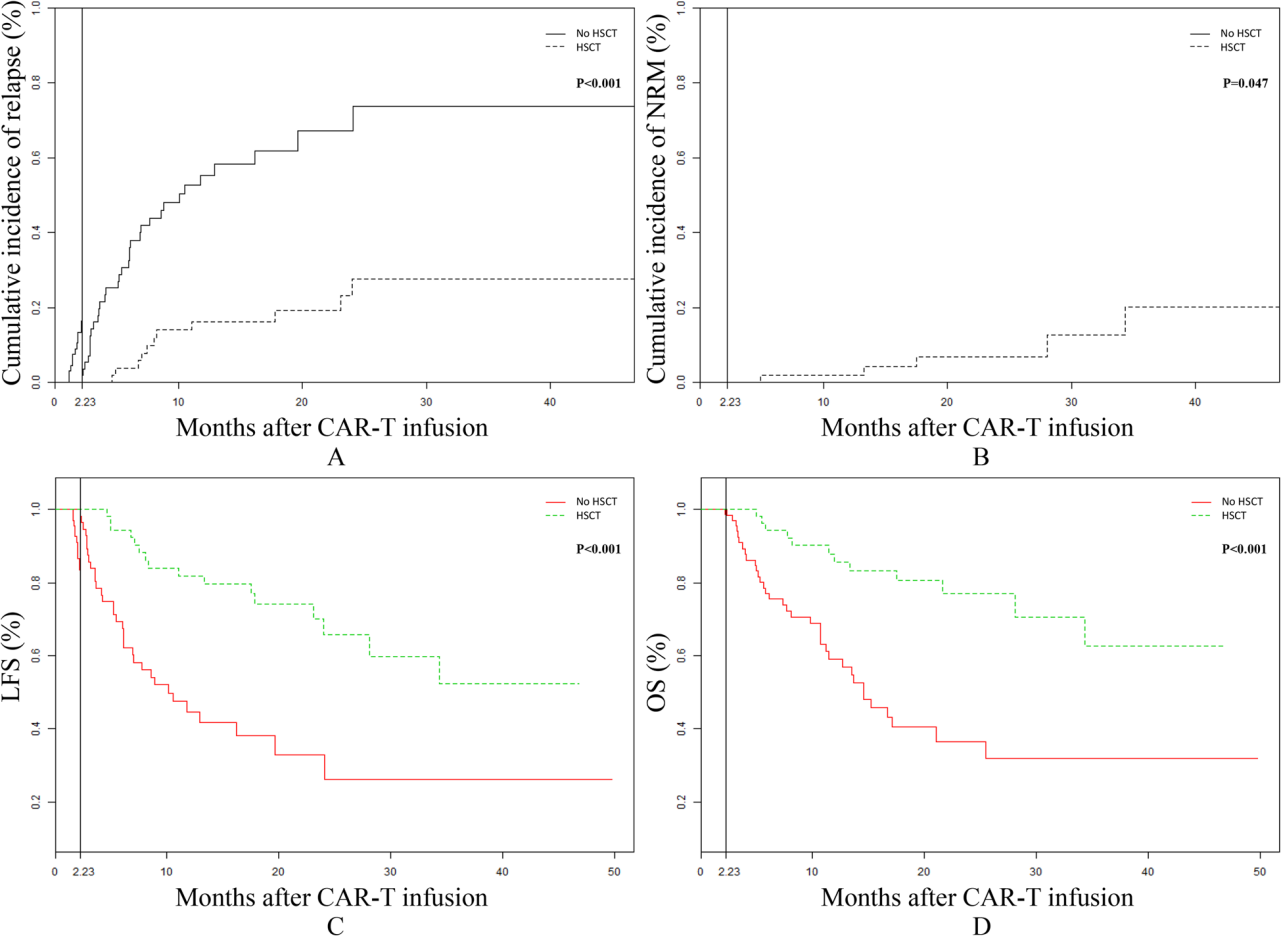
Landmark analysis, cumulative incidence of relapse, non-relapse mortality, leukemia-free survival (LFS) and overall survival (OS) between the non-transplant group and the transplant group. The cut-off value was set at the median time from CAR-T cell infusion to haplo-HSCT (2.23 months). No HSCT: non-transplant group, HSCT: transplant group

Up to the last follow-up, in the non-transplant group, 44 (65.7%) patients relapsed, whereas 11 (20.0%) patients relapsed with CD19 negativity. The 2-year cumulative incidence of relapse in the non-transplant group and transplant group was 67.2% and 27.6% (*P* < 0.001), respectively (Fig. 2a). The 2-year cumulative incidence of NRM in the non-transplant group and transplant group was 0 and 6.8% (*P* = 0.047), respectively (Fig. 2b). The 2-year LFS and OS of patients were higher in the transplant group than in the non-transplant group (65.6% versus 32.8% and 77.0% versus 36.4%, respectively; *P* < 0.001; Fig. 2c and d).

### Factors associated with clinical outcomes

Hazard ratios of prognostic factors associated with LFS, OS, and cumulative incidence of relapse obtained using univariate and multivariate analysis are shown in Supplementary Tables 3-5 and Table 2. MRD positivity at the time of haplo-HSCT (HR: 4.466, 95% CI: 1.561-12.776; *P* = 0.005) and age beyond or equal to 40 years before CAR-T therapy (HR: 4.706, 95% CI: 1.6301–13.586; *P* = 0.004) are the two independent prognostic factors associated with poor LFS. MRD positivity at the time of haplo-HSCT (HR: 3.699, 95% CI: 1.095–12.498; *P* = 0.035) and age beyond or equal to 40 years (HR: 7.110, 95% CI: 2.178–23.210; *P* = 0.001) were two independent prognostic factors associated with poor OS. MRD positivity at the time of haplo-HSCT (HR: 4.190, 95% CI: 1.032–17.013; *P* = 0.045) and more than one relapse before CAR-T infusion (HR: 4.450, 95% CI: 1.069–18.527; *P* = 0.040) were two independent prognostic factors associated with higher cumulative incidence of relapse. For NRM, there were only 5 cases after transplantation which was not adequate for statistical analysis. Complex karyotype, high risk by genetics, primary refraction to chemotherapy, prior lines of chemotherapy, prior allo-HSCT, donor age, and blast cells in the bone marrow before CAR-T infusion were not significant prognostic factors for LFS, OS, or cumulative incidence of relapse.

**Table 2:** Multivariable analyses for factors impacting LFS, OS, and cumulative incidence of relapse in patients who received CAR-T therapy followed by haplo-HSCT

Factors		Hazard ratio (95% CI)	*P* value
LFS			
Age	≥ 40 vs. < 40	4.706 (1.630-13.586)	**0.004**
MRD before HSCT	Positive vs. negative	4.466 (1.561-12.776)	**0.005**
OS			
Age	≥40 vs. <40	7.110 (2.178-23.210)	**0.001**
MRD before HSCT	Positive vs. negative	3.699 (1.095-12.498)	**0.035**
Cumulative incidence of relapse			
Total number of relapses before CAR-T	> 1 vs. ≤ 1	4.450 (1.069-18.527)	**0.040**
MRD before HSCT	Positive vs. negative	4.190 (1.032-17.013)	**0.045**

### Minimal residual disease negativity and favorable outcomes

**Fig. 3:**
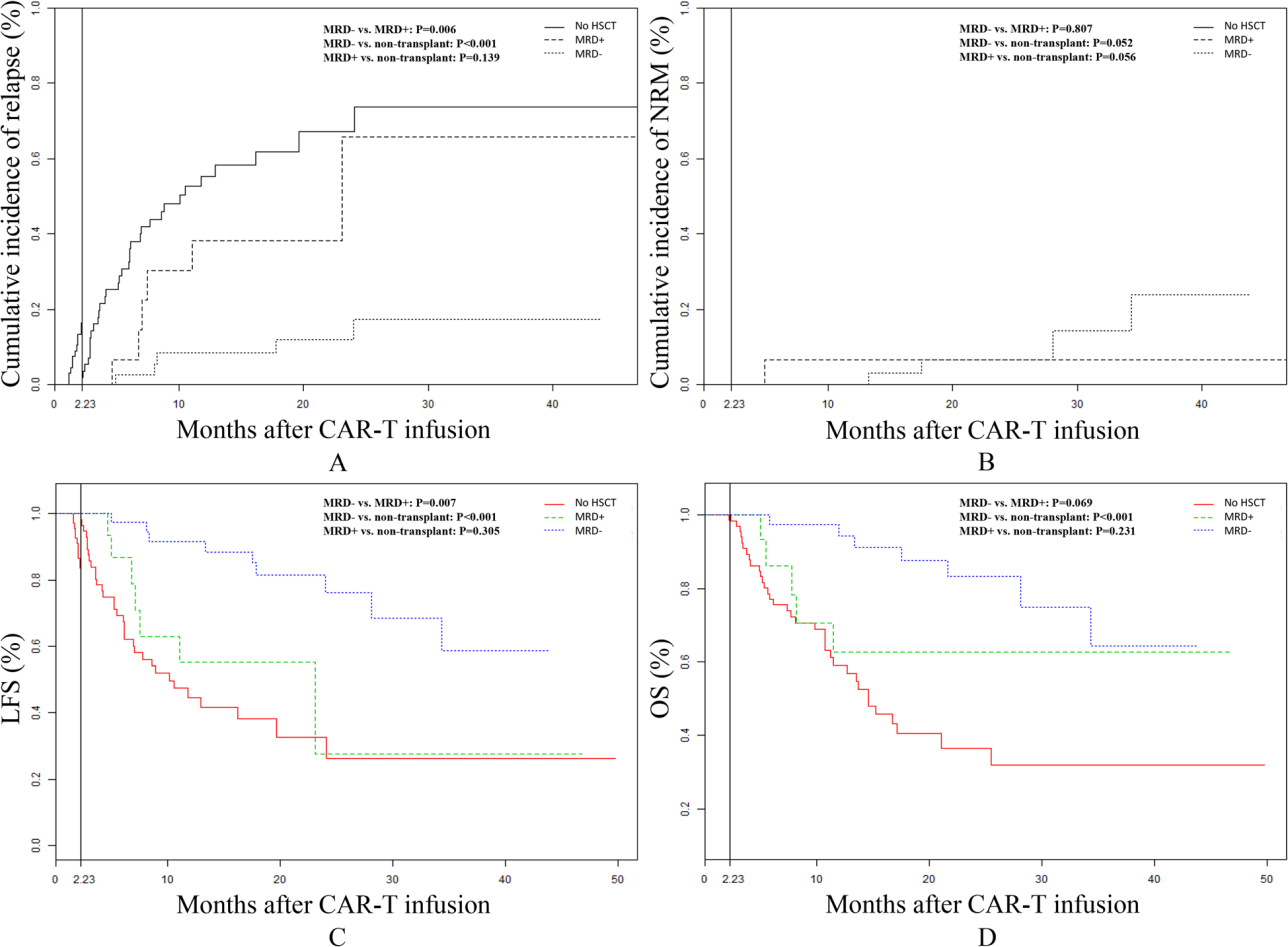
Landmark analysis, cumulative incidence of relapse, non-relapse mortality, leukemia-free survival (LFS) and overall survival (OS) among the non-transplant group, MRD+ group and MRD- group. The cut-off value was set at the median time from CAR-T cell infusion to haplo-HSCT (2.23 months). No HSCT: non-transplant group, MRD+: MRD+ group, MRD-: MRD- group

Patients with pre-transplant MRD negativity and MRD positivity were divided into MRD− group and MRD+ group, respectively. The 2-year cumulative incidence of relapse was 67.2%, 65.8%, and 17.3% in the non-transplant group, MRD+ group, and MRD− group, respectively. Patients in the MRD− group had a lower cumulative incidence of relapse than those in the non-transplant group and in MRD+ group (*P* < 0.001); the cumulative incidence of relapse in patients in the MRD+ group and non-transplant group did not differ significantly (*P* = 0.139; Fig. 3a). The 2-year cumulative incidence of NRM in the MRD− group did not differ from that in the non-transplant group or MRD+ group (6.6% versus 0 and 6.6% versus 6.7%, respectively; *P* = 0.052and 0.807, respectively; Fig. 3b).

The 2-year LFS in the non-transplant group, MRD+ group, and MRD- group was 32.8%, 27.6%, and 76.1%, respectively (Table 3). Patients in the MRD− group had a higher LFS than those in the non-transplant group (*P* < 0.001) and MRD+ group (*P* = 0.007); LFS in the MRD+ group and non-transplant group did not differ significantly (*P* = 0.305) (Fig. 3c). The 2-year OS in the MRD− group was higher than that in the non-transplant group (83.3% versus 36.4%; *P* < 0.001) but did not differ from that in the MRD+ group (83.39% versus 62.7%; *P* = 0.069); the 2-year OS in the non-transplant group and MRD+ group did not differ significantly (*P* = 0.231; Fig. 3d; Table 3).

**Table 3:** Results of main clinical outcomes after CAR-T

Group	1-year cumulative incidence of relapse (%)	2-year cumulative incidence of relapse (%)	1-year LFS (%)	2-year LFS (%)	1-year OS (%)	2-year OS (%)
Non-transplant	55.3	67.2	44.7	32.8	59.1	36.4
MRD+	38.2	65.8	55.2	27.6	62.7	62.7
MRD−	8.4	17.3	91.6	76.1	94.3	83.3
*P between non-transplant and MRD+*		0.139		0.305		0.231
*P between non-transplant and MRD−*		< 0.001		< 0.001		< 0.001
*P between MRD+ and MRD−*		0.006		0.007		0.069

### Engraftment and chimerism after haplo-HSCT

Fifty-five patients who received haplo-HSCT at a median time of 67 days after CAR-T cell infusion (range 34–345 days) were included. The median follow-up time was 613 days after CAR-T therapy (range 100–1403 days). Baseline characteristics at the time of haplo-HSCT are summarized in Table 4. Patients in the MRD+ and MRD− groups were well matched, and there were no significant differences between the two cohorts in terms of underlying disease, time from CAR-T infusion to haplo-HSCT, type of graft, conditioning regimen, GVHD prophylactic regimen, or CD34+ cell dose. After myeloid recovery, all patients achieved sustained, full donor chimerism by day +30 after haplo-HSCT. The median time to neutrophils ≥ 0.5◊10^9^/L after transplantation was 13 days (10–21 days), and the median time to platelets ≥ 20◊10^9^/L after transplantation was 16 days (9–65 days). Two patients suffered from primary platelet dysfunction.

**Table 4:** Patient characteristics among 3 groups

				*P*		
Characteristics	**Non-transplant (*n*= 67)**	**MRD+ (*n*= 15)**	**MRD− (*n*= 40)**	**Non-transplant vs MRD+**	**Non-transplant vs MRD−**	**MRD− vs MRD+**
Gender, *n* (%)				0.112	0.353	0.360
Male	34 (50.7)	11 (73.3)	24 (60.0)			
Female	33 (49.3)	4 (26.7)	16 (40.0)			
Age, years				0.384	0.248	0.931
Median (range)	27.0 (9.0, 65.0)	26.0 (7.0, 65.0)	26.5 (3.0, 63.0)			
Hyperploidy, *n* (%)	3 (4.5)	1 (6.7)	2 (5.0)	0.722	0.901	0.808
Hypoplodiy, *n* (%)	1 (1.5)	0 (0)	1 (2.5)	0.634	0.710	0.537
Complex karyotype, *n* (%)	4 (6.0)	0 (0)	2 (5.0)	0.332	0.833	0.378
iAMP 21^a^, *n* (%)	1 (1.5)	1 (6.7)	0 (0)	0.240	0.438	0.099
ETV6-RUNX1, *n* (%)	1 (1.5)	0 (0)	0 (0)	0.634	0.438	NA
E2A-PBX1, *n* (%)	1 (1.5)	0 (0)	0 (0)	0.634	0.438	NA
MYC mutation, *n* (%)	1 (1.5)	0 (0)	0 (0)	0.634	0.438	NA
HOX11 mutation, *n* (%)	1 (1.5)	0 (0)	0 (0)	0.634	0.438	NA
KMT2A rearranged, *n* (%)	3 (4.5)	1 (6.7)	4 (10.0)	0.722	0.264	0.702
BCR-ABL1, *n* (%)	11 (16.4)	3 (20.0)	8 (20.0)	0.739	0.639	1.000
Ph like, *n* (%)	1 (1.5)	1 (6.7)	3 (7.5)	0.240	0.113	0.916
IgH rearranged, *n* (%)	1 (1.5)	0 (0)	0 (0)	0.634	0.438	NA
IKZF1 mutation, *n*%	3 (4.5)	3 (20.0)	4 (10.0)	**0.037**	0.264	0.322
Poor-risk cytogenetics^b^, *n* (%)	18 (26.9)	6 (40.0)	15 (37.5)	0.312	0.249	0.865
Good-risk cytogenetics^c^, *n* (%)	2 (3.0)	0 (0)	1 (2.5)	0.492	0.883	0.462
Primary refractory to chemotherapy, *n* (%)	17 (25.4)	7 (46.7)	14 (35.0)	0.101	0.288	0.428
Total number of relapses before CAR-T				0.148	0.120	0.633
Median (range)	1 (0, 6)	1 (0, 2)	1 (0, 3)			
Prior lines of therapy				0.519	0.491	0.250
Median (range)	4 (2, 15)	4 (2, 9)	3 (2, 9)			
Prior allo-HSCT, *n* (%)	22 (32.8)	2 (13.3)	1 (2.5)	0.133	**< 0.001**	0.115
Extramedullary infiltration, *n* (%)	9 (13.4)	2 (13.3)	1 (2.5)	0.992	0.060	0.115
Platelet count before CAR-T, 10^9^/L (*N* = 66 + 14 + 38)				0.511	0.186	0.799
Median (range)	130 (15, 412)	139 (12, 302)	140 (21, 389)			
PLT < 1*LLN before CAR-T, n (%) (*N* = 66 + 14 + 38)	28 (42.4)	4 (26.7)	11 (27.5)	0.337	0.172	0.979
LDH > 1*ULN before CAR-T, *n* (%) (*n* = 66 + 14 + 38)	26 (39.4)	6 (40.0)	19 (47.5)	0.810	0.293	0.647
Blast cells in bone marrow before CAR-T				0.802	0.867	0.731
Median (range)	22.5% (0, 97.0%)	6.6% (0.1%, 89.4%)	22.3% (0, 90.0%)			
CAR-T cell dose, 10^6^/kg				0.340	0.302	0.881
Median (range)	3.0 (0.3, 25.0)	5.4 (1.0, 11.1)	5.0 (0.1, 30.4)			
Source of CAR-T cells, *n* (%)				0.438	**0.046**	0.208
Autologous	51 (76.1)	12 (80.0)	38 (95.0)			
Recipient-derived allogenic	9 (13.4)	2 (13.3)	1 (2.5)			
Donor-derived allogenic	6 (9.0)	0 (0)	0 (0)			
Third-party	1 (1.5)	1 (6.7)	1 (2.5)			

^a^*iAMP21* intrachromosomal amplification of chromosome 21

^b^Poor-risk cytogenetics are defined as hypodiploidy, KMT2A rearranged, t(v;14q23)/IgH rearranged, t(9;22)(q34;q11.2): BCR-ABL1, complex karyotype, Ph-like, and iAMP21

^c^Good-risk cytogenetics are defined as hyperdiploidy, and t(12;21)(p13;q22): ETV6-RUNX1

### Acute and chronic graft-versus-host-disease

Supplementary Table 6 summarizes the complications after transplantation. The 100-day cumulative incidence of grade III-IV aGVHD was 7.3% (Supplementary Figure 1A). The 2-year cumulative incidence of cGVHD requiring systemic steroid therapy was 25.5% (Supplementary Figure 1B).

### Infection complications after transplantation

The percentages of patients experiencing at least one bacterial and one invasive fungal infection were 14.0% (8/57) and 5.3% (3/57), respectively. Bacterial infections with *Escherichia coli* (*n* = 2), *Enterococcus faecium* (*n* = 2), *Pseudomonas aeruginosa* (*n* = 2), *Gemella haemolysans* (*n* = 1), *Stenotrophomonas maltophilia* (*n* = 1), *Nocardia farcinica* (*n* = 1), and *Staphylococcus haemolyticus* (*n* = 1) were recorded. All patients responded to antibiotics. Viral infection was also recorded in this population. The 2-year cumulative incidence of CMV and EBV viremia was 56.1% and 57.5%, respectively (Supplementary Figure 1C and D, Supplementary Table 6).

### Other complications after transplantation

Seven patients suffered from cystitis. Two patients developed EBV-associated post-transplant lymphoproliferative disorders and received rituximab with rapid response. One patient developed seizures during the conditioning period. One patient developed immune thrombocytopenia 4 months after transplantation but was refractory to steroids. This patient then received intravenous high-dose immunoglobulin therapy and had a rapid remission.

## Discussion

At present, patients with R/R ALL have a low likelihood of being cured with salvage regimens or available investigational agents. CAR-T therapy has emerged as a rescue for induction therapy in patients with R/R ALL, and clinical studies have reported favorable outcomes in those cases [15–17]. However, relapse after CAR-T treatment remarkably decreases the long-term LFS and OS. As reported, allo-HSCT after CAR-T therapy has the potential to reduce the risk of relapse and improve long-term LFS and OS. Nevertheless, up to date, limited data are available on the clinical outcomes of this combination strategy. In this study, for the first time to our knowledge, we retrospectively reviewed data from CAR-T therapy alone or CAR-T therapy followed by haplo-HSCT for R/R B-ALL in 11 domestic centers in China. Our results showed that patients could greatly benefit from haplo-HSCT after CAR-T therapy. Further analysis indicated that the OS and LFS of patients who received CAR-T treatment followed by haplo-HSCT and achieved pre-transplant MRD negativity tend to be higher than those treated with CAR-T therapy without allo-HSCT, or those who received CAR-T treatment followed by haplo-HSCT and showed pre-transplant MRD positivity. For patients receiving CAR-T treatment followed by haplo-HSCT, MRD negativity at the time of haplo-HSCT was a significant prognostic factor associated with higher LFS and OS. In addition to the beneficial long-term outcomes, risks of treatment-related toxicities were not increased.

Patients with R/R ALL who achieved MRD negativity after chemotherapy and subsequently underwent allo-HSCT are reported to have the best clinical outcomes [18, 19]. Pavlů et al. reported that compared with pre-transplant MRD negativity, pre-transplant MRD positivity was associated with significantly lower OS (61% versus 67%) and LFS (50% versus 58%), and with a higher cumulative incidence of relapse (32% versus 24%) at 2 years post transplantation [20]. In this study, all patients were in the second or third complete remission (CR2 or CR3) or were primary refractory; the 2-year OS, LFS, and cumulative incidence of relapse for patients with pre-transplant MRD negativity and MRD positivity were 83.3% versus 62.7%, 76.1% versus 27.6%, and 17.3% versus 65.8%, respectively. The significantly high OS, LFS, and low incidence of relapse suggest that MRD negativity after CAR-T therapy followed by haplo-HSCT is an effective option for patients with R/R ALL. Several studies have reported that intermediate or high risk identified through risk stratification at diagnosis acts as a significant risk factor for OS and NRM in patients receiving allo-HSCT [21, 22]. Remarkably, multivariate analysis of our data showed that not only risk stratification at diagnosis but also several classic risk factors (such as adverse cytogenetics, total number of relapses, or high leukocyte counts) were no longer associated with poor OS or LFS, which may be related to patient selection and therapy decisions.

Notably, to the best of our knowledge, the present study is the first report of a combined CAR-T and haplo-HSCT strategy against high tumor burden of R/R ALL. CAR-T therapy contributing to pre-transplant MRD negativity plays an important role in favorable clinical outcomes. Haplo-HSCT as consolidation also has a positive effect. In general, haplo-HSCT could have the following advantages for patients: (1) a theoretically high donor availability of almost 100%, (2) a less time-consuming process of finding a donor, and (3) a superior graft-versus-leukemia (GVL) effect [23, 24]. In our previous prospective study, we developed a protocol for T cell-replete haplo-HSCT with low-dose anti-T-lymphocyte globulin and showed that high-risk patients receiving haplo-HSCT experienced protection against relapse. Our protocol provided clinical evidence supporting haplo-identical donors as first-line alternative donors, especially for high-risk patients [25], in line with studies at other centers [26, 27]. Because all patients undergoing CAR-T treatment are relapsed/refractory with high-risk cytogenetics and/or molecular abnormalities, in the present study, we developed a novel strategy using CAR-T cells for re-induction followed by haplo-HSCT for consolidation in patients with R/R ALL. Theoretically, CD19-targeted CAR-T cells would eradicate all CD19-positive leukemia cells; however, we cannot exclude the possibility that certain CD19-negative leukemia sub-clones exist. Considering the results of this study, CAR-T cytotoxicity and GVL effect could be attenuated by conditioning therapy. The synergistic effects of CAR-T and conditioning therapy would potentially eradicate leukemia cells. Hay et al. reported that for patients who received CAR-T therapy to achieve MRD negativity before allo-HSCT, the 2-year LFS and OS were 61% and 72%, respectively. The 2-year cumulative incidence of relapse was 17%^7^, implying a prolonged LFS and OS. The present study showed similar results supporting a combined efficacy of CAR-T therapy and haplo-HSCT. Our results also support that haploidentical immune cells exert a potent GVL effect in such a combination modality.

Recently, studies in adult patients with Ph-negative ALL have established that the initial MRD response is a strong prognostic factor. The German Multicenter Study Group for Adult ALL analyzed the largest cohort of adult ALL data to assess MRD in Ph-negative patients and reported that molecular response was the only parameter with a significant prognostic effect [28]. Other studies confirmed a strong and independent prognostic effect of MRD after induction and early consolidation treatment [29–31]. In the present study, we clearly identified pre-transplant MRD negativity as an important independent predictor of high LFS and OS. Our data support a beneficial modality of haplo-HSCT following CAR-T treatment in patients with R/R ALL.

In addition, we showed that the experience of relapses before CAR-T therapy was another adverse factor for high LFS. In patients with more relapse scenarios, leukemia cells exhibited stronger chemotherapy resistance, more genetic mutations, and immune escape [32, 33]. Moreover, consistent with previous studies in patients who received haplo-HSCT after chemotherapy [34, 35], our study found that age over 40 years was an independent risk factor associated with poor OS [36].

Safety issues are major concerns associated with combination therapy including immunotherapy and HSCT. In the present study, we observed no increased risk of treatment-related complications or immune toxicities. Moreover, no patient died of treatment-related complications. The 100-day cumulative incidence of grade III-IV aGVHD was less than 10% in our study, similar to previously reported values [8, 17, 37]. Allo-HSCT after CAR-T treatment does not seem to increase the risk of therapy-associated complications.

Infection was another severe complication despite CAR-T treatment or allo-HSCT. Park et al. reported that 22 of 53 adult patients experienced 26 infections within the first 30 days after CAR-T infusion, and three patients died of an infection-related cause [38]. Infection is a primary or contributing cause of death in more than half of patients who die in the follow-up period after allo-HSCT [39]. Slade et al. reported that 62% and 6% of patients experienced at least one bacterial and one invasive fungal infection, respectively [39], whereas in the present study, the rates were 14.0% and 5.3%, respectively. Moreover, CMV viremia was detected in the present study and in another study [40]. Our results show that despite the severe CRS and long-term duration of pancytopenia, the novel protocol of CAR-T treatment combined with haplo-HSCT did not increase the risk of infection.

The present study has several limitations, including the retrospective nature, lack of common prospective transplant protocols among the reporting transplant centers, and limited sample size, which may affect the reliability of the statistical analysis. The choice of covariates for the multivariate analysis was constrained by the small number of observed events.

## Conclusions

The present study illustrates the safety and efficacy profiles of a novel combination therapy strategy against R/R ALL by using the combination of CAR-T cells for re-induction followed by haplo-HSCT for consolidation. We confirmed that achieving pre-transplant MRD negativity after CAR-T treatment is a suitable basis for haplo-HSCT. Our results suggest that CAR-T therapy followed by haplo-HSCT could further improve LFS and OS without increasing risks of treatment-related toxicities in a previously heavily treated population. Further research in randomized case-controlled studies with longer follow-up periods is required.

## Supplementary information

**Additional file 1: Supplementary Table S1.** The case number enrolled by each clinical center. **Table S2.** CAR-T therapy associated toxicities. **Table S3.** Univariate analyses for factors impacting LFS in patients who received CAR-T therapy followed by haplo-HSCT. **Table S4.** Univariate analyses for factors impacting OS in patients who received CAR-T therapy followed by haplo-HSCT. **Table S5.** Univariate analyses for factors impacting cumulative incidence of relapse in patients who received CAR-T therapy followed by haplo-HSCT. **Table S6.** Transplant-associated complications. **Figure S1.** Cumulative incidence of grade III-IV acute graft versus host disease (aGVHD), chronic graft versus host disease (cGVHD) requiring systemic steroid therapy, CMV viremia and EBV viremia in transplant group.

## Data Availability

Bone Marrow Transplantation Center, The First Affiliated Hospital, School of Medicine, Zhejiang University will share the study protocol and individual, deidentified participant data that underlie the results reported in this article. The data will be available beginning 9 months and ending 36 months following article publication. The data will be shared with investigators whose proposed use of the data has been approved by Bone Marrow Transplantation Center, The First Affiliated Hospital, School of Medicine, Zhejiang University. The data may be used for individual participant data meta-analysis. Proposals may be submitted up to 36 months following article publication and should be directed to huanghe@zju.edu.cn.

## Abbreviations

aGVHD: Acute graft-versus-host-disease
Allo-HSCT: Allogeneic hematopoietic stem cell transplantation
CAR-T: Chimeric antigen receptor T cell
cGVHD: Chronic graft-versus-host-disease
CI: Confidence intervals
CMV: Cytomegalovirus
CR: Complete remission
CRS: Cytokine release syndrome
EBV: Epstein–Barr virus
GVHD: Graft-versus-host-disease
GVL: Graft versus leukemia
Haplo-HSCT: Haploidentical hematopoietic stem cell transplantation
HR: Hazard ratio
HSCT: Hematopoietic stem cell transplantation
LFS: Leukemia-free survival
MRD: Minimal residual disease
NRM: Non-relapse mortality
OS: Overall survival
Ph: Philadelphia chromosome
R/R ALL: Relapsed/refractory acute lymphoblastic leukemia
