# Supplementary material for: Pre-transplant MRD negativity predicts favorable outcomes of CAR-T therapy followed by haploidentical HSCT for relapsed/refractory acute lymphoblastic leukemia: a multi-center retrospective study
Source: J Hematol Oncol. 2020 May 4;13:42. doi: 10.1186/s13045-020-00873-7 (PMC7199358; doi:10.1186/s13045-020-00873-7)
Supplement: Supplementary file 1 — Additional file 1: Supplementary Table S1. The case number enrolled by each clinical center. Table S2. CAR-T therapy associated toxicities. Table S3. Univariate analyses for factors impacting LFS in patients who received CAR-T therapy followed by haplo-HSCT. Table S4. Univariate analyses for factors impacting OS in patients who received CAR-T therapy followed by haplo-HSCT. Table S5. Univariate analyses for factors impacting cumulative incidence of relapse in patients who received CAR-T therapy followed by haplo-HSCT. Table S6. Transplant-associated complications. Figure S1. Cumulative incidence of grade III-IV acute graft versus host disease (aGVHD), chronic graft versus host disease (cGVHD) requiring systemic steroid therapy, CMV viremia and EBV viremia in transplant group. [file 13045_2020_873_MOESM1_ESM.zip › Supplementary materials.docx]

Supplementary Table 1. The case number enrolled by each clinical center

| Clinical center | Case number (%) |
| --- | --- |
| The First Affiliated Hospital, Zhejiang University | 37 (30.3) |
| Tianjin First Central Hospital | 27 (22.1) |
| Shanghai Tongji Hospital | 14 (11.5) |
| Guangdong Second Provincial General Hospital | 14 (11.5) |
| Changhai Hospital of Shanghai | 10 (8.2) |
| Xiangya Third Hospital | 10 (8.2) |
| Shanghai Children's Medical Center | 3 (2.5) |
| Shanghai General Hospital | 2 (1.6) |
| Peking University Third Hospital | 2 (1.6) |
| Zhujiang Hospital of Southern Medical University | 2 (1.6) |
| Xinhua Hospital of Shanghai | 1 (0.8) |

Supplementary Table 2. CAR-T therapy associated toxicities

|  |  |  |  | *P* |
| --- | --- | --- | --- | --- |
| Characteristics | **ALL (N=122)** | **Non-transplant (N=67)** | **Transplant (N=55)** | **Non-transplant vs Transplant** |
| Time to peak of CRS, days |  |  |  | 0.058 |
| Median (interquartile range) | 6.0 (4.0, 9.0) | 7.0 (3.0, 9.0) | 5.0 (4.0, 8.0) |  |
| IL-6 at peak of CRS, pg/ml |  |  |  | 0.411 |
| Median (interquartile range) | 109.2 (28.8, 864.5) | 98.4 (33.6, 593.9) | 132.2 (28.7, 986.4) |  |
| IL-10 at peak of CRS, pg/ml |  |  |  | 0.784 |
| Median (range) | 46.7 (10.9, 156.5) | 37.4 (8.7, 108.5) | 61.7 (17.4, 261.9) |  |
| IFN-γ at peak of CRS, pg/ml |  |  |  | 0.326 |
| Median (range) | 26.0 (9.2, 388.9) | 15.6 (5.7, 291.0) | 55.8 (9.9, 685.6) |  |
| Grade of CRS, n (%) |  |  |  | 0.602 |
| 0 | 9 (7.4) | 5 (7.5) | 4 (7.3) |  |
| 1 | 46 (37.7) | 27 (40.3) | 19 (34.5) |  |
| 2 | 35 (28.7) | 16 (23.9) | 19 (34.5) |  |
| 3 | 27 (22.1) | 15 (22.4) | 12 (21.8) |  |
| 4-5 | 5 (4.1) | 4 (6.0) | 1 (1.8) |  |
| Platelet count decreases to < 10000/μl related to CAR-T, n (%) | 77 (63.1) | 42 (62.7) | 35 (63.6) | 0.914 |
| Neutrophil count decreases to < 500/μl related to CAR-T, n (%) | 91 (74.6) | 54 (80.6) | 37 (67.3) | 0.093 |
| Time to platelet count < 10000/μl after CAR-T, days |  |  |  | 0.169 |
| Median (interquartile range) | 2.0 (1.0, 6.0) | 2.5 (1.0, 7.3) | 2.0 (1.0, 6.0) |  |
| Time to neutrophil count < 500/μl after CAR-T, days |  |  |  | 0.230 |
| Median (interquartile range) | 2.0 (1.0, 6.0) | 2.0 (0.8, 5.3) | 3.0 (1.0, 7.0) |  |
| Duration of platelet count < 10000/μl after CAR-T, days |  |  |  | 0.215 |
| Median (interquartile range) | 8.0 (3.3, 18.5) | 7.0 (4.0, 20.0) | 9.0 (3.0, 17.0) |  |
| Duration of neutrophil count < 500/μl after CAR-T, days |  |  |  | 0.323 |
| Median (interquartile range) | 5.0 (2.0, 12.0) | 5.0 (2.8, 13.3) | 7.0 (2.0, 11.5) |  |
| Infection confirmed by culture, n (%) | 7 (5.7) | 4 (6.0) | 3 (5.5) | 0.903 |

Supplementary Table 3. Univariate analyses for factors impacting LFS in patients who received CAR-T therapy followed by haplo-HSCT.

| Factors |  | Hazard ratio (95% CI) | P-value |
| --- | --- | --- | --- |
| Sex | Male vs. female | 0.432 (0.160-1.164) | 0.097 |
| Age |  | 1.036 (1.005-1.067) | **0.020** |
| Age | ≥40 vs. <40 | 3.850 (1.378-10.754) | **0.010** |
| KMT2A rearranged | Yes vs. no | 0.773 (0.101-5.922) | 0.804 |
| BCR-ABL1 | Yes vs. no | 1.839 (0.666-5.083) | 0.240 |
| Ph-like | Yes vs. no | 1.896 (0.244-14.735) | 0.541 |
| IKZF1 mutation | positive vs. negative | 1.022 (0.232-4.511) | 0.977 |
| Poor-risk cytogenetics | Yes vs. no | 1.618 (0.606-4.322) | 0.337 |
| Total number of relapses before CAR-T |  | 1.263 (0.672-2.374) | 0.468 |
| Primary refractory to chemotherapy | Yes vs. no | 1.093 (0.393-3.035) | 0.865 |
| Prior lines of chemotherapy |  | 0.921 (0.691-1.229) | 0.577 |
| PLT < 1*LLN before CAR-T | Yes vs. no | 1.316 (0.449-3.860) | 0.617 |
| PLT/LLN before CAR-T |  | 1.099 (0.640-1.887) | 0.732 |
| LDH > 1*ULN before CAR-T | Yes vs. no | 1.570 (0.556-4.435) | 0.395 |
| LDH/ULN before CAR-T |  | 1.046 (0.789-1.386) | 0.755 |
| Blast cells in bone marrow before CAR-T |  | 1.006 (0.989-1.023) | 0.473 |
| Autologous CAR-T | Yes vs. no | 0.182 (0.048-0.686) | **0.012** |
| Infusion dose of CAR-T cells |  | 1.065 (0.992-1.143) | 0.080 |
| MRD at 7-10 day after CAR-T | Positive vs. negative | 1.991 (0.622-6.375) | 0.246 |
| Time to peak of CRS after CAR-T |  | 1.078 (0.901-1.289) | 0.411 |
| Grade of CRS |  | 0.983 (0.576-1.679) | 0.951 |
| Neutropenia related to CAR-T therapy | Yes vs. no | 1.435 (0.461-4.472) | 0.533 |
| Platelet count < 10000/μl related to CAR-T therapy | Yes vs. no | 1.139 (0.393-3.306) | 0.810 |
| Time from CAR-T to HSCT | Yes vs. no | 1.003 (0.997-1.009) | 0.316 |
| MRD before HSCT | Positive vs negative | 3.698 (1.340-10.205) | **0.012** |
| Maternal hemapoietic stem cell donor | Yes vs. no | 0.778 (0.102-5.962) | 0.809 |
| Paternal hemapoietic stem cell donor | Yes vs. no | 0.419 (0.133-1.324) | 0.138 |
| Sibling hemapoietic stem cell donor | Yes vs. no | 1.190 (0.382-3.703) | 0.764 |
| Offspring hemapoietic stem cell donor | Yes vs. no | 2.162 (0.798-5.861) | 0.130 |
| Age of hemapoietic stem cell donor |  | 0.996 (0.960-1.034) | 0.844 |
| Sex of hemapoietic stem cell donor | Male vs. female | 0.565 (0.198-1.610) | 0.285 |
| Infusion dose of CD34+ cells |  | 1.071 (0.867-1.322) | 0.524 |
| The date of platelet engraftment |  | 0.982 (0.919-1.048) | 0.584 |
| The date of granulocyte engraftment |  | 0.958 (0.773-1.186) | 0.692 |
| Infectious fever during haplo-HSCT | Yes vs. no | 1.347 (0.428, 4.237) | 0.610 |

Supplementary Table 4. Univariate analyses for factors impacting OS in patients who received CAR-T therapy followed by haplo-HSCT.

| Factors |  | Hazard ratio (95% CI) | P-value |
| --- | --- | --- | --- |
| Sex | Male vs. female | 0.390 (0.124-1.233) | 0.109 |
| Age |  | 1.044 (1.010-1.079) | **0.010** |
| Age | ≥40 vs. <40 | 5.873 (1.877-18.374) | **0.002** |
| KMT2A rearranged | Yes vs. no | 1.122 (0.142-8.881) | 0.913 |
| BCR-ABL1 | Yes vs. no | 0.963 (0.260-3.573) | 0.955 |
| Ph-like | Yes vs. no | 3.261 (0.404-26.287) | 0.267 |
| IKZF1 mutation | positive vs. negative | 0.639 (0.082-4.958) | 0.668 |
| Poor-risk cytogenetics | Yes vs. no | 1.116 (0.354-3.522) | 0.851 |
| Total number of relapses before CAR-T |  | 1.379 (0.684-2.782) | 0.369 |
| Primary refractory to chemotherapy | Yes vs. no | 1.293 (0.406-4.112) | 0.664 |
| Prior lines of chemotherapy |  | 0.972 (0.710-1.332) | 0.861 |
| PLT < 1*LLN before CAR-T | Yes vs. no | 0.948 (0.250-3.596) | 0.938 |
| PLT/LLN before CAR-T |  | 1.299 (0.693-2.435) | 0.415 |
| LDH > 1*ULN before CAR-T | Yes vs. no | 1.973 (0.575-6.765) | 0.280 |
| LDH/ULN before CAR-T |  | 1.005 (0.700-1.444) | 0.978 |
| Blast cells in bone marrow before CAR-T |  | 1.009 (0.990-1.028) | 0.350 |
| Infusion dose of CAR-T cells |  | 1.058 (0.975-1.149) | 0.177 |
| MRD at 7-10 day after CAR-T | Positive vs. negative | 1.737 (0.459-6.574) | 0.416 |
| Time to peak of CRS after CAR-T |  | 1.147 (0.932-1.411) | 0.196 |
| Grade of CRS |  | 0.739 (0.396-1.379) | 0.342 |
| Neutropenia related to CAR-T therapy | Yes vs. no | 2.602 (0.570-11.877) | 0.217 |
| Platelet count < 10000/μl related to CAR-T therapy | Yes vs. no | 1.570 (0.423-5.822) | 0.500 |
| Time from CAR-T to HSCT | Yes vs. no | 0.999 (0.990-1.008) | 0.782 |
| MRD before HSCT | Positive vs negative | 2.813 (0.879-8.999) | 0.081 |
| Maternal hemapoietic stem cell donor | Yes vs. no | 1.169 (0.148-9.261) | 0.882 |
| Paternal hemapoietic stem cell donor | Yes vs. no | 0.453 (0.121-1.696) | 0.240 |
| Sibling hemapoietic stem cell donor | Yes vs. no | 0.601 (0.131-2.749) | 0.511 |
| Offspring hemapoietic stem cell donor | Yes vs. no | 2.939 (0.932-9.268) | 0.066 |
| Age of hemapoietic stem cell donor |  | 1.006 (0.963-1.050) | 0.799 |
| Sex of hemapoietic stem cell donor | Male vs. female | 0.719 (0.213-2.431) | 0.596 |
| Infusion dose of CD34+ cells |  | 1.142 (0.903-1.444) | 0.269 |
| The date of platelet engraftment |  | 0.968 (0.881-1.063) | 0.490 |
| The date of granulocyte engraftment |  | 0.967 (0.756-1.237) | 0.789 |
| Infectious fever during haplo-HSCT | Yes vs. no | 1.450 (0.391, 5.382) | 0.578 |

Supplementary Table 5. Univariate analyses for factors impacting cumulative incidence of relapse in patients who received CAR-T therapy followed by haplo-HSCT.

| Factors |  | Hazard ratio (95% CI) | P-value |
| --- | --- | --- | --- |
| Sex | Male vs. female | 0.544 (0.160-1.855) | 0.331 |
| Age |  | 1.035 (0.991-1.081) | 0.119 |
| Age | ≥40 vs. <40 | 2.166 (0.548-8.565) | 0.271 |
| KMT2A rearranged | Yes vs. no | 1.316 (0.157-11.010) | 0.800 |
| BCR-ABL1 | Yes vs. no | 1.229 (0.310-4.874) | 0.769 |
| Ph-like | Yes vs. no | 3.261 (0.404-26.287) | NA |
| IKZF1 mutation | positive vs. negative | 0.749 (0.093-6.053) | 0.786 |
| Poor-risk cytogenetics | Yes vs. no | 0.818 (0.229-2.927) | 0.757 |
| Total number of relapses before CAR-T |  | 2.196 (1.044-4.620) | **0.038** |
| Total number of relapses before CAR-T | >1 vs. ≤1 | 5.190 (1.467-18.363) | **0.011** |
| Primary refractory to chemotherapy | Yes vs. no | 0.462 (0.116-1.846) | 0.275 |
| Prior lines of chemotherapy |  | 0.828 (0.582-1.179) | 0.296 |
| PLT < 1*LLN before CAR-T | Yes vs. no | 0.924 (0.235-3.632) | 0.910 |
| PLT/LLN before CAR-T |  | 1.056 (0.579-1.924) | 0.860 |
| LDH > 1*ULN before CAR-T | Yes vs. no | 0.924 (0.257-3.322) | 0.904 |
| LDH/ULN before CAR-T |  | 1.155 (0.829-1.609) | 0.394 |
| Blast cells in bone marrow before CAR-T |  | 1.005 (0.984-1.026) | 0.645 |
| Infusion dose of CAR-T cells |  | 0.966 (0.815-1.145) | 0.693 |
| MRD at 7-10 day after CAR-T | Positive vs. negative | 1.197 (0.239-5.994) | 0.827 |
| Time to peak of CRS after CAR-T |  | 1.041 (0.838-1.295) | 0.716 |
| Grade of CRS |  | 1.646 (0.759-3.566) | 0.207 |
| Grade of CRS | >1 vs. ≤1 | 1.130 (0.318-4.008) | 0.851 |
| Grade of CRS | >2 vs. ≤2 | 4.547 (1.211-17.066) | **0.025** |
| Neutropenia related to CAR-T therapy | Yes vs. no | 1.435 (0.375-5.494) | 0.598 |
| Platelet count < 10000/μl related to CAR-T therapy | Yes vs. no | 1.169 (0.328-4.166) | 0.810 |
| Time from CAR-T to HSCT | Yes vs. no | 0.998 (0.991-1.005) | 0.588 |
| MRD before HSCT | Positive vs negative | 4.755 (1.381-16.370) | **0.013** |
| Maternal hemapoietic stem cell donor | Yes vs. no | 0.624 (0.082-4.758) | 0.649 |
| Paternal hemapoietic stem cell donor | Yes vs. no | 0.607 (0.155-2.381) | 0.473 |
| Sibling hemapoietic stem cell donor | Yes vs. no | 2.605 (0.723-9.391) | 0.143 |
| Offspring hemapoietic stem cell donor | Yes vs. no | 0.806 (0.201-3.226) | 0.761 |
| Age of hemapoietic stem cell donor |  | 1.019 (0.977-1.064) | 0.382 |
| Sex of hemapoietic stem cell donor | Male vs. female | 0.965 (0.276-3.372) | 0.955 |
| Infusion dose of CD34+ cells |  | 1.437 (1.048-1.970) | **0.024** |
| Infusion dose of CD34+ cells | ≥6 vs. <6 | 5.466 (1.531-19.513) | **0.009** |
| The date of platelet engraftment |  | 0.974 (0.900-1.054) | 0.517 |
| The date of granulocyte engraftment |  | 0.962 (0.780-1.185) | 0.713 |
| Infectious fever during haplo-HSCT | Yes vs. no | 2.714 (0.572-12.881) | 0.209 |

Supplementary Table 6. Transplant-associated complications

|  |  |  |  | *P* |
| --- | --- | --- | --- | --- |
| Complication | **Transplant (n=55)** | **MRD+ (n=15)** | **MRD- (n=40)** | **MRD+ vs MRD-** |
| aGVHD, n (%) |  |  |  | 0.467 |
| 0 | 20 (36.4) | 6 (40.0) | 14 (35.0) |  |
| I | 24 (43.6) | 8 (53.3) | 16 (40.0) |  |
| II | 7 (12.7) | 0 (0) | 7 (17.5) |  |
| III | 3 (5.5) | 1 (3.7) | 2 (5.0) |  |
| IV | 1 (1.8) | 0 (0) | 1 (2.5) |  |
| cGVHD, n (%) |  |  |  | 0.515 |
| 0 | 33 (60.0) | 11 (73.3) | 22 (55.0) |  |
| Mild | 9 (16.4) | 2 (13.3) | 7 (17.5) |  |
| Moderate | 10 (18.2) | 1 (6.7) | 9 (22.5) |  |
| Severe | 3 (5.5) | 1 (6.7) | 2 (5.0) |  |
| Infection confirmed by culture | 9 (16.4) | 1 (6.7) | 8 (20.0) | 0.234 |
| Viral infection |  |  |  |  |
| CMV | 30 (54.5) | 8 (53.3) | 22 (55.0) | 0.912 |
| EBV | 30 (54.5) | 8 (53.3) | 22 (55.0) | 0.912 |
| Other virus |  |  |  |  |
| BK virus | 15 (27.3) | 2 (13.3) | 13 (32.5) | 0.155 |
| JC virus | 6 (10.9) | 2 (13.3) | 4 (10.0) | 0.724 |
| Herpes virus | 2 (3.6) | 0 (0) | 2 (5.0) | 0.378 |
| Influenza virus | 3 (5.5) | 2 (13.3) | 1 (2.5) | 0.115 |
| Parvovirus | 2 (3.6) | 1 (6.7) | 1 (2.5) | 0.462 |
| Cystitis | 7 (12.7) | 2 (13.3) | 5 (12.5) | 0.934 |
| Seizures | 1 (1.8) | 1 (6.7) | 0 (0) | 0.099 |
| EBV associated PTLD | 2 (3.6) | 0 (0) | 2 (5.0) | 0.378 |
| Primary platelet dysfunction | 2 (3.6) | 0 (0) | 2 (5.0) | 0.378 |
| ITP | 1 (1.8) | 0 (0) | 1 (2.5) | 0.537 |
| Gastrointestinal hemorrhage | 1 (1.8) | 1 (6.7) | 0 (0) | 0.099 |
| Autoimmune hemolytic anemia | 1 (1.8) | 0 (0) | 1 (2.5) | 0.537 |

**Supplementary Figure 1** Cumulative incidence of grade Ⅲ-Ⅳ acute graft versus host disease (aGVHD), chronic graft versus host disease (cGVHD) requiring systemic steroid therapy, CMV viremia and EBV viremia in transplant group.
