# Supplementary figures and images for: Pre-transplant MRD negativity predicts favorable outcomes of CAR-T therapy followed by haploidentical HSCT for relapsed/refractory acute lymphoblastic leukemia: a multi-center retrospective study
Source: J Hematol Oncol. 2020 May 4;13:42. doi: 10.1186/s13045-020-00873-7 (PMC7199358; doi:10.1186/s13045-020-00873-7)

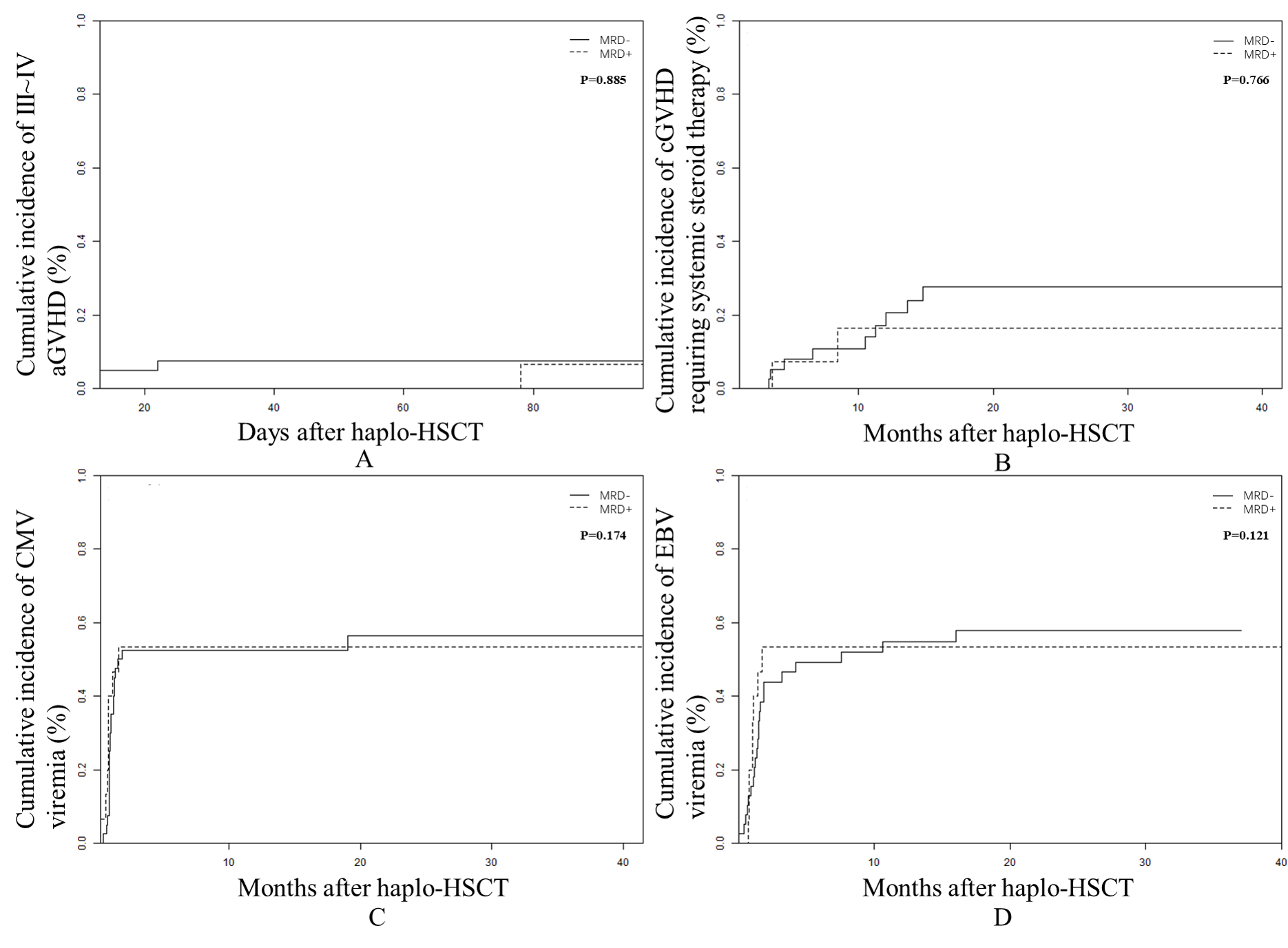

Supplement: Supplementary file 1 — Additional file 1: Supplementary Table S1. The case number enrolled by each clinical center. Table S2. CAR-T therapy associated toxicities. Table S3. Univariate analyses for factors impacting LFS in patients who received CAR-T therapy followed by haplo-HSCT. Table S4. Univariate analyses for factors impacting OS in patients who received CAR-T therapy followed by haplo-HSCT. Table S5. Univariate analyses for factors impacting cumulative incidence of relapse in patients who received CAR-T therapy followed by haplo-HSCT. Table S6. Transplant-associated complications. Figure S1. Cumulative incidence of grade III-IV acute graft versus host disease (aGVHD), chronic graft versus host disease (cGVHD) requiring systemic steroid therapy, CMV viremia and EBV viremia in transplant group. [file 13045_2020_873_MOESM1_ESM.zip › supplementary figure.png]
